# Integrative ATAC-seq and RNA-seq Analysis of the Longissimus Muscle of Luchuan and Duroc Pigs

**DOI:** 10.3389/fnut.2021.742672

**Published:** 2021-09-29

**Authors:** Weiwei Miao, Zeqiang Ma, Zhanyang Tang, Lin Yu, Siqi Liu, Tengda Huang, Peng Wang, Tian Wu, Ziyi Song, Haojie Zhang, Yixing Li, Lei Zhou

**Affiliations:** ^1^State Key Laboratory for Conservation and Utilization of Subtropical Agro-Bioresources, College of Animal Science and Technology, Guangxi University, Nanning, China; ^2^Tilapia Seed Farm, Guangxi Academy of Fishery Sciences, Nanning, China

**Keywords:** ATAC-seq, RNA-seq, transcription factor, longissimus dorsi, Luchuan, Duroc

## Abstract

Luchuan pig is a typical obese pig breed in China, and the diameter and area of its longissimus dorsi muscle fibers are significantly smaller than those of Duroc (lean) pig. Skeletal muscle fiber characteristics are related to meat quality of livestock. There is a significant correlation between the quality of different breeds of pork and the characteristics of muscle fiber, which is an important factor affecting the quality of pork. The diameter and area of muscle fibers are related to muscle growth and development. Therefore, we used the assay for transposase-accessible chromatin using sequencing (ATAC-seq) and RNA sequencing (RNA-seq) analysis to investigate the potential mechanism underlying the difference in skeletal muscle growth and development between the two types of pigs. First, transposase-accessible chromatin was analyzed to map the landscape of open chromatin regions and transcription factor binding sites. We identified several transcription factors that potentially affected muscle growth and development, including TFAP4, MAX, NHLH1, FRX5, and TGIF1. We also found that transcription factors with basic helix-loop-helix structures had a preference for binding to genes involved in muscle development. Then, by integrating ATAC-seq and RNA-seq, we found that the Wnt signaling pathway, the mTOR signaling pathway, and other classical pathways regulate skeletal muscle development. In addition, some pathways that might regulate skeletal muscle growth, such as parathyroid hormone synthesis, secretion, and action, synthesis and degradation of ketone bodies, and the thyroid hormone signaling pathway, which were significantly enriched. After further study, we identified a number of candidate genes (*ASNS, CARNS1, G0S2, PPP1R14C*, and *SH3BP5*) that might be associated with muscle development. We also found that the differential regulation of chromatin openness at the level of some genes was contrary to the differential regulation at the level of transcription, suggesting that transcription factors and transcriptional repressors may be involved in the regulation of gene expression. Our study provided an in-depth understanding of the mechanism behind the differences in muscle fibers from two species of pig and provided an important foundation for further research on improving the quality of pork.

## Introduction

Porcine skeletal muscle is one of the main sources of protein for the Chinese people, and skeletal muscle accounts for 40% of the body weight (1). The development and growth of skeletal muscle determine the quality and yield of meat (2). With the improvement of people's living standards, meat of increasingly high quality is pursued, and the meat from Duroc and other lean pigs can no longer meet the people's needs. Previous researchers have found that meat quality was related to muscle fiber characteristics and intramuscular fat content (3, 4). The character of muscle fiber is an important factor affecting meat quality (5, 6). One reason is that muscle fiber size affects the size of the muscle bundle, resulting in coarser muscle transverse sections (7, 8). Another reason is that the size of muscle fiber is related to tenderness; the smaller the muscle fiber, the lower the shear force, the better the tenderness and thus the better the meat quality (6). The shear force of pork is related to tenderness. Some researchers found that the meat quality of Lantang pig was better than Landrace pig. The shear force of Lantang pig was 61.23N, which significantly lower than that of Landrace pig (78.8N) (9). Luchuan pig is a typical fat breed, which is characterized by good meat quality, high reproductive performance, and a slow growth rate (10). Meanwhile, the diameter and area of the longissimus dorsi muscle fibers are significantly smaller than those of Duroc pigs. They are good models for studying the molecular mechanism underlying differences in meat quality. Therefore, studies on the differences between the longissimus dorsi muscle fibers of Luchuan and Duroc pigs can provide a direction for improving meat quality.

The muscle fiber diameter is related to muscle growth and development. It has been reported that the growth and development of skeletal muscle were regulated by interactions of multiple functional genes. The roles of transcription factors of the myogenic regulatory factor (MRF) family (11) and the myocyte enhancer factor-2 (MEF2) family (12) in the development of skeletal muscle have been intensively studied. Members of the MRF family, including myogenic differentiation antigen (MYOD), myogenic factor 5 (MYF5), myogenin (MYOG), and myogenic factor 6 (MYF6), which are key inducers of skeletal muscle development (13), are characterized by basic helix-loop-helix (bHLH) structures. The MEF2 family also has four members, namely MEF2a, MEF2b, MEF2c, and MEF2d. They all share an N-terminal MADS domain and a binding domain of MEF2, which can specifically bind A/T-enriched conserved sequences (14). There is a direct interaction between the MEF2 gene family and the MRF gene family (15). They can regulate each other's expression through the bHLH and MADS domains, and in most cases, the activation of genes related to muscle development is regulated synergistically. Many classical pathways such as the Notch signaling pathway (16), the Wnt signaling pathway (17), and the mTOR signaling pathway (18) are all key pathways for muscle proliferation and differentiation. In addition, some hormones such as parathyroid hormone (19) and thyroid hormone (20) also regulate skeletal muscle development. Recently, some researchers have studied the skeletal muscle development at different embryonic stages of the same pig breed from the perspective of chromatin openness and RNA sequencing (RNA-seq). However, the molecular mechanisms underlying differences in muscle growth between breeds remain unclear. Particularly, the molecular mechanisms underlying differences in meat quality have not been analyzed from the perspective of chromatin openness characteristics.

In this study, we performed an assay for transposase-accessible chromatin using sequencing (ATAC-seq) (21) for the detection of open chromatin regions of the longissimus dorsi muscle in two pig species in order to identify transcription factors that regulate muscle growth and development. By comparing ATAC-seq results with RNA-seq data, we further identified a number of potential core genes that regulated muscle proliferation and differentiation. In addition, some pathways that regulated this process were identified. These results will provide a useful resource for further exploration of the mechanisms underlying differences in meat quality.

## Materials and Methods

### Animals and Sample Collection

Three Luchuan and three Duroc boars were used in this study. Animals had free access to food and water and were kept under the same conditions. All pigs were put to death at the age of 180 days, with overnight fasting prior to death. Samples of the longissimus dorsi muscle were collected and frozen in liquid nitrogen for subsequent experiments. All animal experiments were approved by the Institutional Animal Care and Use Committee of Guangxi University (GXU-2015-003).

### Histological Analysis

To investigate the size of muscle fibers, the muscle was immobilized in 4% paraformaldehyde solution for 24 h and completely embedded in paraffin. A solidified paraffin block was cut crosswise and stained with hematoxylin and eosin (H&E). Histological examination was conducted under a light microscope. Three tissue sections of each pig were included, and five visual fields were randomly selected for each section under 40× magnification. Images were collected using an upright microscope. At least 50 muscle fibers were randomly selected by Image-Pro Plus 6.0 Image analysis software, and the diameter and area of muscle fibers in the collected images were measured.

### ATAC-seq

The original Illumina high-throughput sequencing image data files were converted into original sequences after base group recognition by bcl2fastq software (22). The results were stored in FASTQ file format. Trimmomatic (v0.36) software was used to filter the original sequences to obtain high quality clean reads, and Bowtie2 software was used to compare the sequencing sequences with the reference genome (Sus scrofa 11.1) (23). MACS2 software was used for peak detection to obtain an overview of the open chromatin regions of the whole genome of each sample (24). Different peaks were identified based on the following criteria: |log_2_(fold change)| ≥ 1 and *P* < 0.00001. The genes corresponding to the different peaks of each sample were identified and annotated. Three biological replicates were used. The motif was examined using the Multiple EM for Motif Elicitation (MEME) suite and transcription factors were predicted using the motif database scanning algorithm TOMTOM (25).

### RNA-seq

Transcriptome data were assembled using StringTie and compared with the reference genome (Sus scrofa 10.2) using CuffCompare (26). The calculated gene expression was directly used to compare gene expression between different samples. Difference analysis software DEGseq was used to analyze the differences between groups. Significantly differentially expressed genes (DEGs) were identified based on the following criteria: |log_2_(fold change)| ≥ 1 and *q* < 0.001. Three biological replicates were used.

### Integration Analysis of ATAC-seq and RNA-seq

The genes associated with the open chromatin regions in Duroc and Luchuan overlapped with the up and down regulated genes in the Duroc and Luchuan transcriptomes, respectively.

### Gene Functional Annotation

Gene Ontology (GO) enrichment analysis of DEGs was performed using topGO software. KOBAS software was used for Kyoto Encyclopedia of Genes and Genomes (KEGG) pathway enrichment analysis among DEGs.

### Analysis of Gene Expression

Three duplicate tissue samples were obtained from each pig. Total RNA was isolated using TRIzol reagent (OMEGA, USA; Genstar, Beijing, China) and then reverse transcribed using a first strand cDNA synthesis kit (Takara, Dalian, China) according to the manufacturer's instructions. The primers used in this study are shown in Table 1. Thermal cycling conditions were as follows: 95°C for 1 min, followed by 40 cycles of 10 s at 95°C, 34 s at 60°C, and 1 min at 60°C. Three biological replicates were used. Primers for selected genes were designed by primer 5.0. Cycle threshold values were collected and normalized to that of *TBP*.

**Table 1:** qPCR primer sequence.

**List**	**Name**	**Primer sequence (5′ to 3′)**
1	PVALB_F	AGGAGGAGGAGCTGGGATTC
2	PVALB_R	AGCTTTCAGCCACCAGAGTG
3	LGMN_F	AGACGCTCCACAAACAGTAC
4	LGMN_R	CAACTTCATGGCAGAGATGGA
5	PPP1R14C_F	CACCAGCAAGGAAAAGTGACAG
6	PPP1R15C_R	GCATTTCTTCTTCCTCGCAGC
7	EGF_F	TCTGAACCCGGACGGATTTG
8	EGF_R	GACATCGCTCGCGAACGTAG
9	PDK4_F	ACCAGGAAAACTGGCCTTCT
10	PDK4_R	GGAACACCACCTCCTCTGTC
11	ASNS_F	GTGTTCAGAAGCTAAAGGTCTCGTT
12	ASNS_R	GGCGACTTTGCCATTTGG
13	G0S2_F	GTCGCCTTACGTTTGGACTTGC
14	G0S2-R	CAGGTAACTCCGCTCAGGTGC
15	CCNYL1_F	AGCTGAGTTGGATTACGGAGC
16	CCNYL1_R	TCCTCTTTTCTCGCACATCTGTT
17	TBP_F	GAACTGGCGGAAGTGACGTT
18	TBP_R	GCACAGCAAGAAAGAGTGATGC

### Statistics

All data are expressed as mean ± SEM. Statistical analysis was performed by the unpaired two-tailed Student's *t*-test. The probability level of *P* < 0.05 (^*^) or *P* < 0.01 (^**^) is used to indicate significance.

## Results

### Phenotype and ATAC-seq Quality Control of the Duroc and Luchuan Pig Muscle Tissues

**Figure 1:**
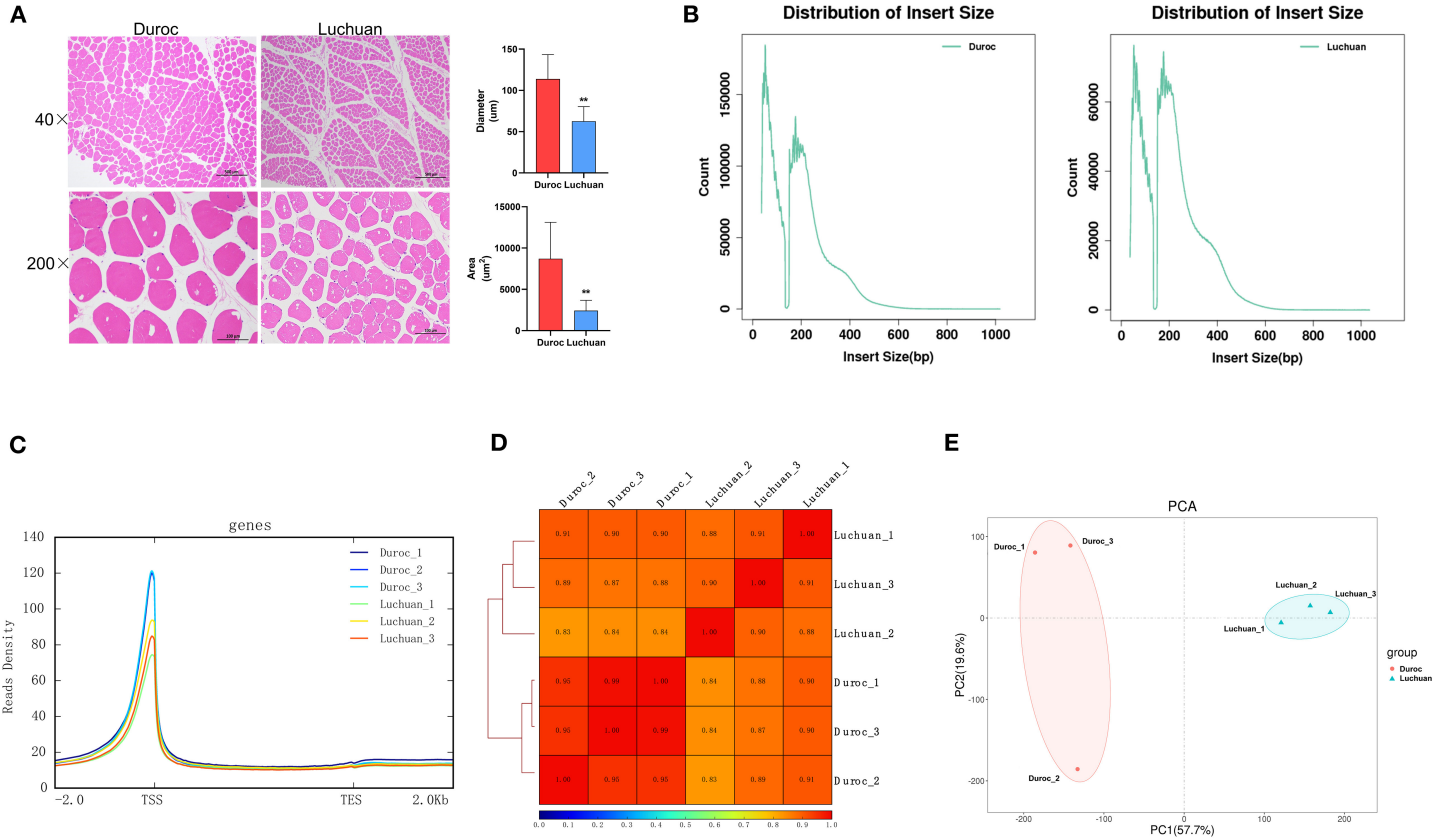
Phenotype and ATAC-seq quality control. **(A)** Muscle tissue sections at 40× and 200× magnification. Quantification of the diameter and area of muscle fibers. The data are expressed as mean ± SD. **P < 0.01. **(B)** Distribution of insert size. **(C)** Enrichment of ATAC-seq signals around the TSS. The *x*-axis represents the normalized gene or peak length, and the *y*-axis represents the read enrichment. The larger the value, the higher the enrichment. TSS, transcription start site; TES, transcription end site. −2.0 represents 2 kb upstream of the TSS, and 2.0 represents 2 kb downstream of the TES. **(D)** The Pearson's correlation results shown by a heat map scatterplot. **(E)** Principal component analysis (PCA).

It is well-known that the meat quality of Luchuan pigs is higher than that of Duroc pigs, partially because the diameter and area of muscle fiber of Luchuan pigs are smaller than those in Duroc pigs (Figure 1A).

To unveil the mechanism behind the muscle fiber difference, ATAC-seq was performed to investigate the chromatin accessibility of the genome. A total of 767,777,278 raw reads were obtained. After filtration, 723,124,192 clean reads were obtained (Table 2). All libraries produced fragment lengths with expected distribution (Figure 1B). The highest peak on the left was a nucleosome-free fragment corresponding to the open chromatin region (the nucleosome cleaved fragment <147), and the highest peak on the right was the open chromatin fragment representing the fragment including two nucleosomes (the nucleosome cleaved fragment >147 and <147 × 2), which is consistent with the characteristics of open region of chromatin revealed by other studies. Most of the identified accessible areas were enriched in 2 kb of transcription start site (Figure 1C), indicating that open regions of chromatin were involved in transcriptional regulation. To further determine the quality of our ATAC-seq data, pairwise Pearson correlation between any pair of ATAC-seq samples was calculated according to the read signals from the combined ATAC-seq peaks of all samples, and the results showed that there were similarities between pigs of the same species and differences between the two breeds (Figure 1D). Principal component analysis (PCA) revealed the similarity between biological replicates and the difference between the two pig breeds (Figure 1E). All these results suggested that the quality of the sequencing data was very high.

**Table 2:** ATAC-seq data statistics.

**Sample**	**Duroc _1**	**Duroc _2**	**Duroc _3**	**Luchuan _1**	**Luchuan _2**	**Luchuan _3**
Raw reads	158,494,044	134,992,362	138,531,738	110,414,912	106,505,090	118,839,132
Raw bases (G)	23.77	20.25	20.78	16.56	15.98	17.83
Raw Q30 (%)	89.72	90.2	88.31	90.26	90.1	90.72
Raw GC (%)	48.8	49.98	48.84	47.72	47.27	49.35
Clean reads	149,338,986	127,614,802	130,236,642	104,104,332	99,337,648	112,491,782
Clean bases (G)	15.97	14.1	13.76	11.75	11.59	12.68
Clean Q30 (%)	96.22	96.03	95.17	96.03	95.82	96.08
Clean GC (%)	48.72	50.2	48.66	47.3	46.98	49.63
Clean reads rate (%)	94.22	94.53	94.01	94.28	93.27	94.66

### Chromatin Accessibility in the Muscle of Duroc and Luchuan Pigs

**Figure 2:**
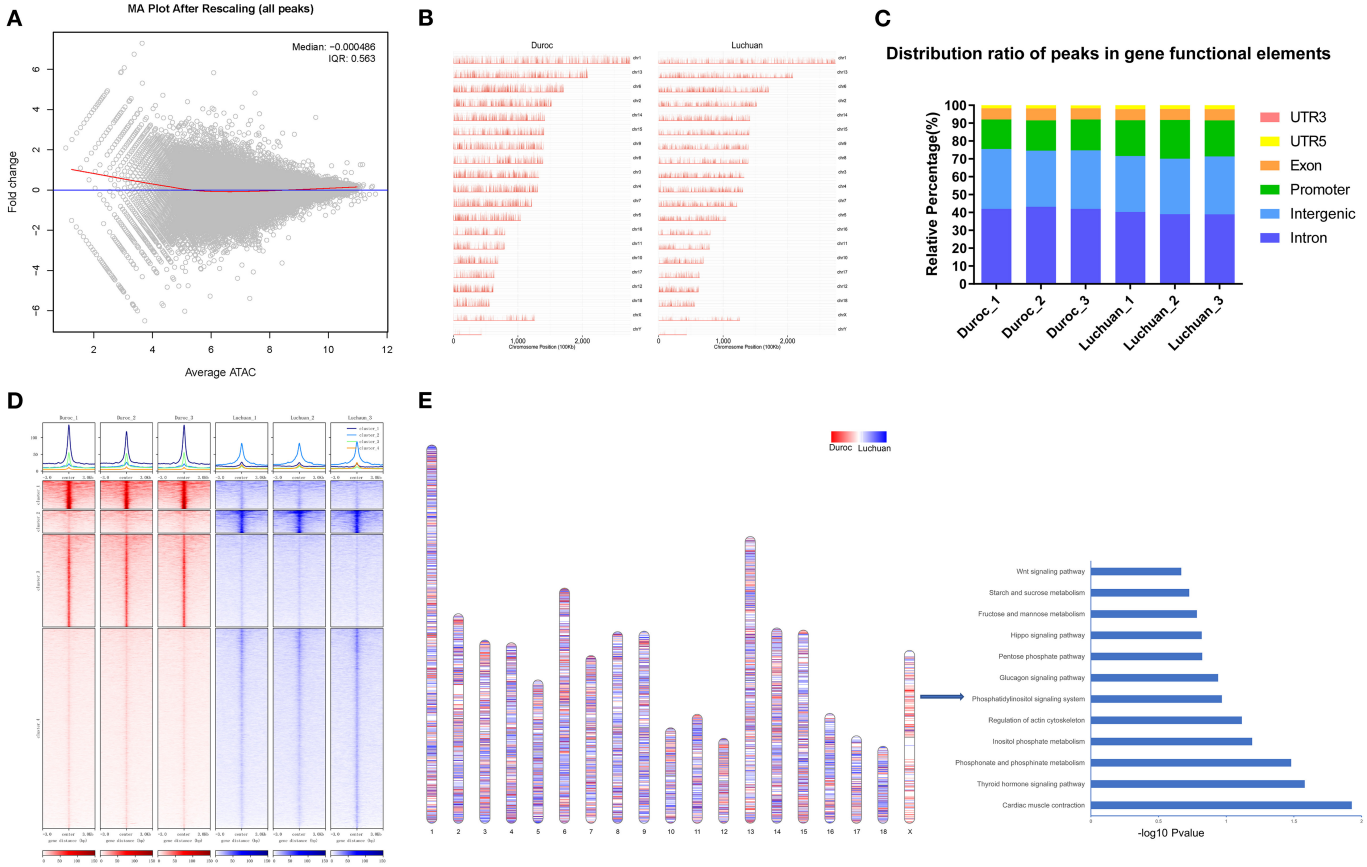
Analyses of the peaks. **(A)** MA plot. The *x*-axis represents signal strength in the open region of chromatin, and the *y*-axis represents fold change. **(B)** Chromosomal distribution of all peaks. **(C)** Distribution ratio of peaks in gene functional elements. **(D)** Top, enrichment of ATAC-seq signals around the 3 kb upper and lower reaches of the peak center. Bottom, peaks are divided into four categories according to the signal intensity. **(E)** Left, chromosome distribution of Duroc peaks and Luchuan peaks (*P* < 0.01). Right, KEGG pathway enrichment analysis of the genes corresponding to Duroc peaks on the X chromosome.

We identified 44,532 peaks specific to Duroc, 26,773 peaks specific to Luchuan, and 68,776 common peaks. The MA plot in Figure 2A shows the relationship between the open intensity and fold change of all peaks. As can be seen from the distribution map of all peaks on the chromosome, the signal intensity of Duroc peaks was higher than that of Luchuan peaks in total (Figure 2B). Genome-wide functional regions are divided into introns, intergenic regions, promoters, exons, 5'UTRs, and 3'UTRs to annotate all peaks (Figure 2B). We found that the peaks of the promoter regions in Luchuan accounted for 20.62% of the total area, while those of Duroc accounted for 16.90% (Figure 2C). As shown in the heat map, the peaks of Duroc and Luchuan were divided into four clusters. The clusters revealed differences in chromatin openness between the two pig breeds (Figure 2D). Compared with cluster1 and cluster2, the signal intensity was not only higher but also had the biggest difference. Compared with cluster3 and cluster4, although the signal intensity was different, the signal intensity was weaker than that of cluster1 and cluster2, which suggested that the key factors that might regulate muscle development were these strong signal peaks. There was a big difference between the genome-wide Duroc peak and Luchuan peak, but whether there was a chromosomal difference is not clear. Thus, the distribution of Duroc peaks and Luchuan peaks on chromosomes (the length of each different peak was standardized according to the length of each chromosome) was further studied. It was found that the peaks of Duroc were much more enriched on the X chromosome than those of Luchuan. It seems that downregulated chromatin accessibility regions most likely enriched in X chromosome. Then, the genes corresponding to downregulated chromatin accessibility regions on chromosome X were subjected to KEGG pathway enrichment analysis (Figure 2E). Our data revealed that regulation of the actin cytoskeleton, the Hippo signaling pathway, and the Wnt signaling pathway were enriched pathways involved in muscle development, which indicated that the X chromosome was an important chromosome that regulated muscle development, which is consistent with other studies (27, 28).

### Corresponding Genes and Motif Analysis in Different Peaks

**Figure 3:**
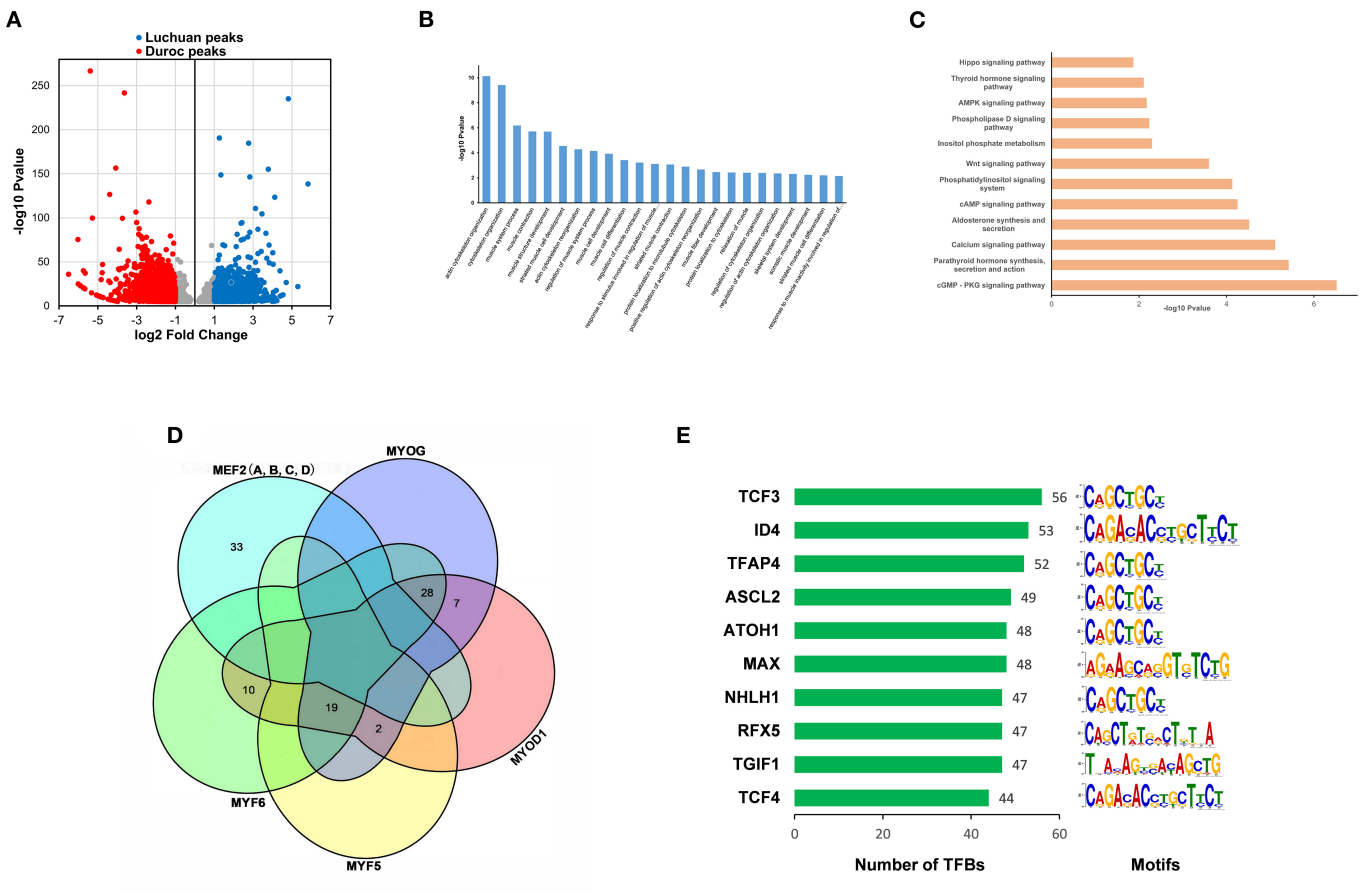
Different peaks corresponding to target gene enrichment analyses and transcription factor prediction. **(A)** Scatter plot of differential peaks (*P* < 0.00001, |log_2_(fold change)| ≥ 1). **(B)** GO enrichment analysis of target genes corresponding to different peaks. **(C)** KEGG pathway enrichment analysis of target genes corresponding to different peaks. **(D)** Unified Venn diagrams of predicted binding sites of star factors (MRFs and MEF2s) involved in the regulation of muscle development according to motif alignment. **(E)** Predicted transcription factors. Left, the number of co-binding sites between the predicted transcription factors and the star factors. Right, the motif type of the transcription factors.

In order to identify the open sites related to phenotype, different peaks were filtered with *P* < 0.00001 |log_2_(fold change)| ≥ 1. There were 3,412 different peaks in duroc pigs, 1,639 different peaks in Luchuan pigs (Figure 3A). By annotating each different peak, the different peaks of Duroc correspond to 1,376 genes, and the different peaks of Luchuan correspond to 628 genes, and a total of 1,905 genes were obtained. GO analysis and KEGG pathway enrichment analysis were conducted with these genes. Pathways with *P* ≤ 0.05 were considered significantly enriched. In the biological process category of GO enrichment, mainly processes related to muscle cell differentiation and muscle development were enriched, among which actin cytoskeleton organization was the most significant (Figure 3B). Moreover, 74 KEGG pathways were significantly enriched (*P* ≤ 0.05), many of which are involved in muscle development, such as the Wnt signaling pathway, the AMPK signaling pathway, and the Hippo signaling pathway (29–31). We showed 12 classical pathways associated with muscle development in Figure 3C. Duroc muscles are better developed than those of Luchuan. On this foundation, we analyzed the motifs on the different peaks of Duroc using the MEME suite and predicted the transcription factors by using the motif database scanning algorithm TOMTOM. Finally, 373 transcription factors (*P* < 0.05) were predicted. The transcription factors MYOD, MYOG, MYF5, MYF6, and members of the MEF2 family were key factors, which regulated the expression of many genes related to muscle development. Based on our data, they may bind to a total of 99 sites in different Duroc peaks (Figure 3D). Interestingly, the MEF2 family did not intersect with the other four transcription factors, suggesting it was regulated to muscle development in different ways. Among all the predicted transcription factors, 160 transcription factors shared common binding sites with these key factors. Sorted by the number of common binding sites, the top 10 transcription factors are shown in Figure 3E. Interestingly, nine of these 10 transcription factors have been reported to be associated or potentially associated with muscle development.

### RNA-seq Data From Duroc and Luchuan Pig Muscle Tissues

High-throughput mRNA sequencing was performed to evaluate the transcriptome. PCA showed that there was a correlation within groups and a difference between groups of mRNA abundance among biological repeats (Supplementary Figure 1A). In total, 22,787 genes were identified in Duroc and 22,778 genes were identified in Luchuan by RNA-seq. The abundance of mRNA in two groups followed an approximately lognormal distribution (Supplementary Figures 1B,C). In total, 21,560 genes were shared by Duroc and Luchuan (Supplementary Figure 1D). The average abundance of mRNA in Duroc had a high correlation with that in Luchuan (*R*^2^ = 0.899; Supplementary Figure 1E).

**Figure 4:**
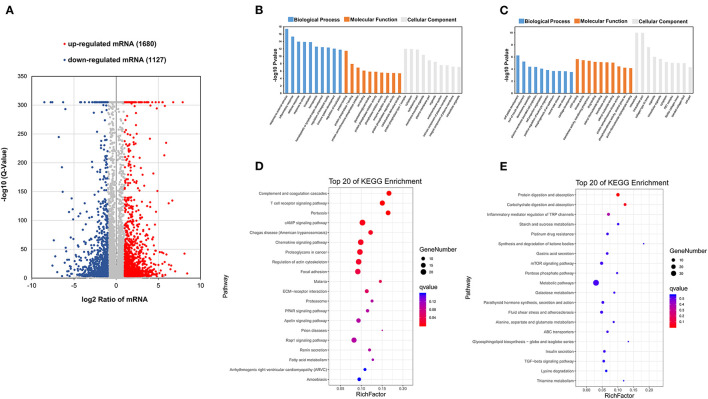
Analyses of RNA-seq. **(A)** Scatter plot of transcriptome data (*q* < 0.001, |log_2_(fold change)| ≥ 1). **(B)** GO enrichment analysis of upregulated mRNAs. **(C)** GO enrichment analysis of downregulated mRNAs. **(D)** KEGG classification of upregulated mRNAs. **(E)** KEGG classification of downregulated mRNAs.

In order to identify the crucial functional genes related to phenotype, differentially expressed mRNAs (DEmRNAs) were filtered based on the criteria |log_2_(fold change)| ≥ 1 and *q* < 0.001. Overall, 1,680 upregulated and 1,127 downregulated mRNAs were screened (Figure 4A). GO analysis and KEGG pathway enrichment analysis were used to analyze these up and downregulated mRNAs. All DEmRNAs were mapped to terms of the GO database, enabling annotation. These upregulated DEmRNAs were enriched in protein-related processes, etc. (Figure 4B). These downregulated DEmRNAs were enriched in ATP binding, etc. (Figure 4C). Some KEGG pathways related to protein metabolism and energy metabolism were significantly enriched. These upregulated DEmRNAs were enriched in cAMP signaling pathway, etc. (Figure 4D). These downregulated DEmRNAs were enriched in protein digestion and absorption, starch and sucrose metabolism, the mTOR signaling pathway, ABC transporters, and the TGF-beta signaling pathway (Figure 4E) (29–31). This suggested that DEmRNAs may be involved in maintaining intramuscular homeostasis and muscle development.

### Integration of ATAC-seq Results With RNA-seq

**Figure 5:**
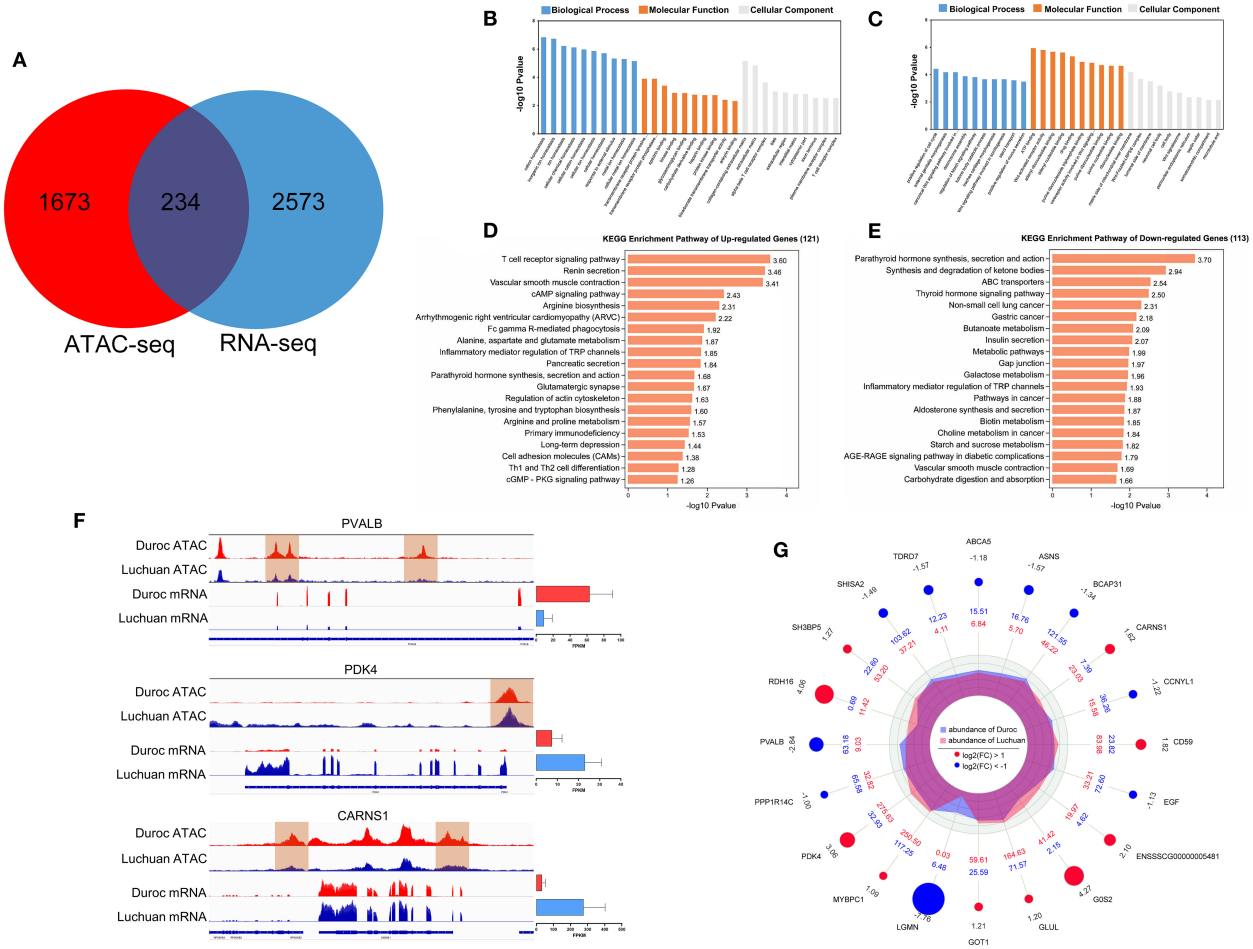
Analysis results of integrated ATAC-seq and RNA-seq results. **(A)** Intersection of different mRNAs and different peaks corresponding to the target genes. **(B)** GO enrichment of upregulated DEGs. **(C)** GO enrichment of downregulated DEGs. **(D)** KEGG classification of upregulated DEGs. **(E)** KEGG classification of downregulated DEGs. **(F)** Peak diagram showing the relationship between genes in open regions of chromatin and gene expression. Among them, the *PVALB* gene of Duroc had a different peak and its transcriptional expression level was high; the *PDK4* gene of Luchuan had a different peak and its transcriptional expression level was high; the *CARNS1* gene of Duroc had different peaks but its transcriptional expression level was low. **(G)** A radar map of the top 20 candidate genes.

To determine whether changes in open chromatin regions in ATAC-seq analysis were associated with changes in gene expression, we integrated ATAC-seq data with RNA-seq data. In total, 234 DEGs were identified (Figure 5A), including 121 upregulated genes and 113 downregulated genes.

According to the GO annotation of these DEGs, it is interesting to note that the 121 upregulated genes were significantly enriched in pathways related to muscle homeostasis and muscle maintenance, including ion homeostasis and cellular homeostasis (Figure 5B). Meanwhile, the 113 downregulated genes were enriched in the Wnt signaling pathway, ATP binding, the Notch signaling pathway, and so on, which are related to muscle development regulation (Figure 5C). After annotating the DEGs to KEGG pathways, the data showed that some pathways related to energy metabolism and protein metabolism, such as the cAMP signaling pathway, galactose metabolism, starch and sucrose metabolism, alanine, aspartate, and glutamate metabolism, and phenylalanine, tyrosine, and tryptophan biosynthesis were significantly enriched (Figures 5D,E). Interestingly, parathyroid hormone synthesis, secretion, and action was the most significantly enriched pathway. In order to further determine the relationship between chromatin openness and gene expression and to further investigate how transcription factors regulate downstream genes, we analyzed the relationship between gene openness and gene expression. As shown in Table 3, 67 genes were upregulated and 17 genes were downregulated in the region with high chromatin openness, while 96 genes were downregulated and 54 genes were upregulated in the region with low chromatin openness. This may be due to the involvement of transcriptional factors or transcriptional repressors in the regulation of gene expression. Some examples are shown in Figure 5F. The chromatin accessibility and transcription level of *PVALB* in Duroc were higher than those in Luchuan. On the contrary, the chromatin accessibility and transcription level of *PDK4* in Luchuan were higher than those in Duroc. Interestingly, the chromatin accessibility of *CARNS1* in Duroc was higher than that in Luchuan, however, the transcription level was lower. According to the *P*-value, we focused on the top 20 DEGs (Figure 5G). Interestingly, nine of the 20 candidate genes have been reported to be associated with muscle development, and five have been reported to be potentially associated with muscle development.

**Table 3:** Chromatin openness of differentially expressed genes.

**Sample**	**ATAC-up**		**ATAC-down**	
	**RNA-up**	**RNA-down**	**RNA-down**	**RNA-up**
GeneNum	67	17	96	54

**Figure 6:**
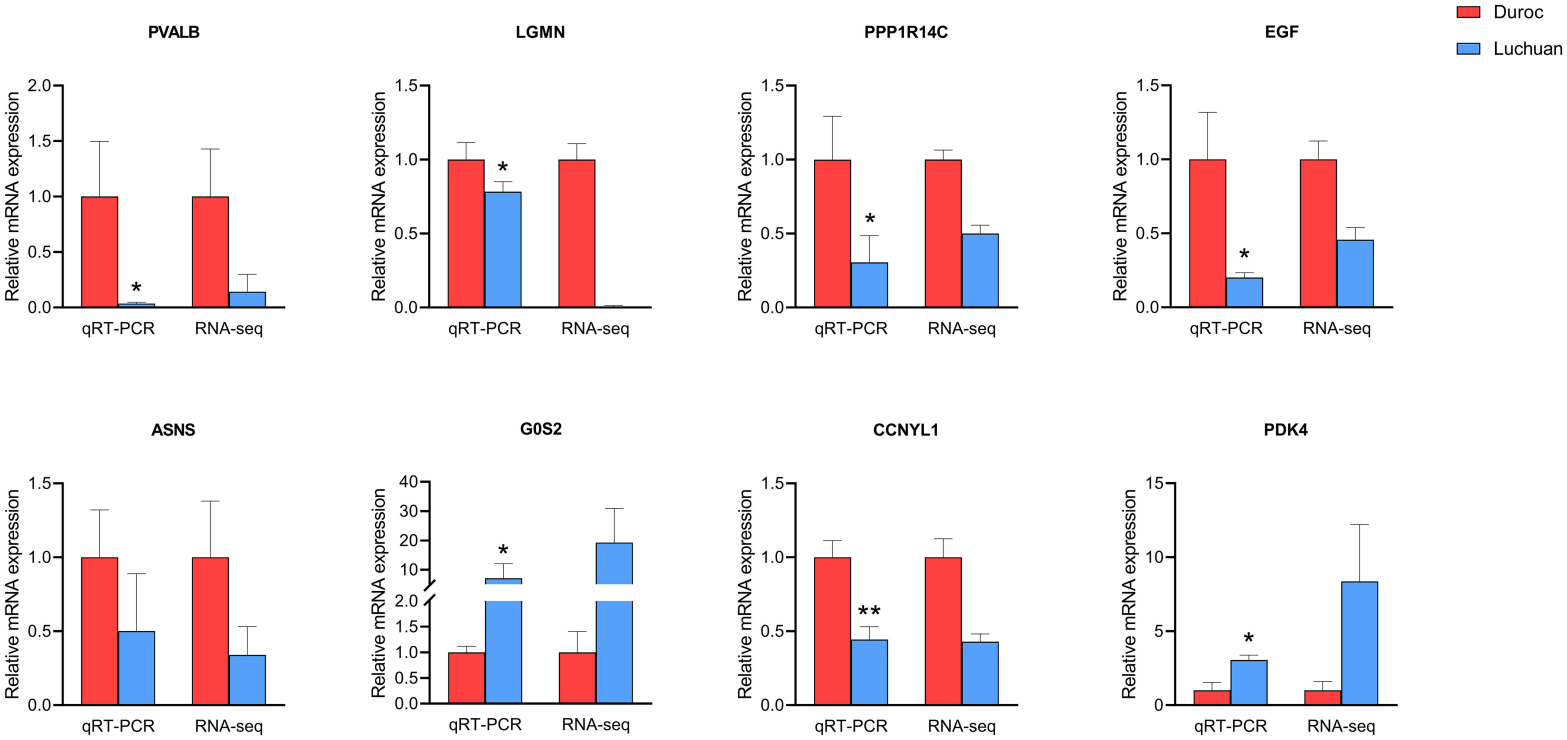
Verification of RNA-seq data by qRT-PCR. The left *y*-axis represents the relative expression level as determined by qRT-PCR, and the right *y*-axis represents the FPKM value as determined by RNA-seq. All data represent the average value of three biological replicates. The error line represents three repeated standard errors, and all data are normalized. *P < 0.05, **P < 0.01.

To validate the accuracy of the RNA-seq data, eight genes randomly selected from the top 20 DEGs (*PVALB, LGMN, PPP1R14C, EGF, ASNS, G0S2, CCNYL1*, and *PDK4*) were analyzed by qRT-PCR. These total RNA samples were taken from the longissimus dorsi muscle of three Duroc and three Luchuan pigs. The gene expression patterns were similar in our RNA-seq and qRT-PCR analyses (Figure 6), which confirmed the accuracy of our RNA-seq data in both pig muscles.

## Discussion

Pig skeletal muscle growth and development is one of the key factors that influence the quality of pork. Genome-wide chromatin accessibility regulate Luchuan pigs and Duroc pigs skeletal muscle growth and development by binding different TFs. The number of open sites and the signal intensity had great difference partly interpret the difference in muscle development between the two pig breeds (32). At the same time, we identified pathways and genes that may regulate the growth and development of skeletal muscle through ATAC-seq and RNA-seq analysis.

ATAC-seq identified three methylated open chromatin regions of H3K4, H3K36, and H3K79 (33). MRF and MEF2 transcription factors were involved in muscle proliferation and differentiation, and they all had a bHLH structure. In general, all bHLH transcription factors have a common motif or domain. Some transcription factors that share common binding sites with MRF and MEF2 families were screened out by the co-localization method. The more common binding sites there were, the more likely they were to have similar functions (34). We focused on the top 10 transcription factors with the most binding sites. Interestingly, these 10 transcription factors all had a bHLH structure, among which four transcription factors, including TCF3, ID4, ASCL2, and TCF4, have already been reported to regulate muscle development. The TCF3 protein forms a heterodimer with muscle regulators such as MYOD, which then binds to DNA and regulates the transcription of target genes related to muscle differentiation (35). ID4 inhibits DNA binding of E47 homotypes and E47/MYOD heterodimers, and is a key transcriptional regulator of muscle-related genes (36). The expression of ASCL2 is regulated by the Notch signal, and inhibits myogenesis by antagonizing the transcriptional activity and blocking the regeneration of injured muscles (37). TCF4 is strongly expressed in connective tissue fibroblasts, and low levels of TCF4 in myoblasts promote slow and rapid myogenesis, thereby promoting the overall maturation of muscle fiber types (38, 39). Besides, five other transcription factors, including TFAP4 (40), MAX (41, 42), NHLH1 (43), FRX5 (44, 45), and TGIF1 (46, 47), have potential roles in muscle development. Whether ATOH1 is involved in muscle development is still unclear.

The integration of ATAC-seq and RNA-seq results showed that 121 genes were upregulated and 113 genes were downregulated in Luchuan vs. Duroc. We focused on the top 20 genes with transcriptome *P*-values. Nine of these genes, including *BCAP31* (48), *CD59* (49), *EGF* (50), *GLUL* (51), *GOT1* (52), *MYBPC1* (53), *PDK4* (54), *PVALB* (55), and *SHISA2* (56), have been reported to be involved in muscle development. Five genes, namely *ASNS* (57), *CARNS1* (58), *G0S2* (59), *PPP1R14C* (60, 61), and *SH3BP5* (62), have been reported to be potentially associated with muscle development. For six other genes, including *ABCA5, CCNYL1, LGMN, ENSSSCG00000005481, RDH16*, and *TDRD7*, so far there are no reports that showed a link with muscle development, thus their function in muscle development and function remains to be explored in the future.

Skeletal muscle development is regulated by various signaling pathways. Our KEGG pathway analysis results revealed some classical pathways that participated in the regulation of muscle development (Figures 5D,E). In addition, we found that some hormones, such as parathyroid hormone and thyroid hormone, may also regulate muscle development, because organs involved in calcium and phosphorus metabolism are related to parathyroid hormone, which directly responds to myocyte factors (19). Skeletal muscle is the primary target of thyroid hormone signaling and thyroid hormone signaling is involved in important biological functions, including energy consumption, heat production, development and growth (20). These findings provided new direction for further exploring meat quality.

Furthermore, ATAC-seq and RNA-seq integration analysis showed that not all genes with increased chromatin openness had increased gene expression. Some genes showed the opposite result, which may be caused by the binding of transcription repressors to the open chromatin area (63). Our data indicated that chromatin openness was not directly correlated with gene expression. The specific reasons remain to be further studied.

## Conclusion

Overall, in this study we present a novel method for identifying regions of open chromatin and predicting transcription factors (TFAP4, MAX, NHLH1, FRX5, and TGIF1), involved in the regulation of muscle development in different breeds of pigs. With the combination of ATAC-Seq and RNA-Seq, we identified several candidate genes (*ASNS, CARNS1, G0S2, PPP1R14C*, and *SH3BP5*) that may regulate muscle development. In addition to this, we found that muscle development was maybe related to some of the hormonal signaling pathways (parathyroid hormone synthesis and action and thyroid hormone signaling pathway) in two breeds of pigs. This study can therefore be used as a reference for future research on the meat quality differences between Luchuan and Duroc pigs.

## Data Availability Statement

The datasets presented in this study can be found in online repositories. The names of the repository/repositories and accession number(s) can be found below: NCBI GEO; GSE180840.

## Ethics Statement

All animal experiments were approved by the Institutional Animal Care and Use Committee of Guangxi University (GXU-2015-003).

## Author Contributions

WM, ZM, and LZ conceived the project, designed the protocol, and wrote the manuscript. ZT, LY, SL, and TH analyzed the data. PW, TW, ZS, HZ, and YL performed the experiments. All authors have read and approved the final manuscript.

## Funding

This work was supported by grants from the National Key R&D Program of China (2018YFD0500402), Guangxi Science Foundation for Distinguished Young Scholars (2020GXNSFFA297008), Guangxi Science and Technology Base and Talents Project (AD18281085), Guangxi Natural Science Foundation (2019GXNSFDA245029), Guangxi Hundred-Talent Program, State Key Laboratory for Conservation and Utilization of Subtropical Agro-bioresources (SKLCUSA-a202006), and Training Project of High-level Professional and Technical Talents of Guangxi University.

## Conflict of Interest

The authors declare that the research was conducted in the absence of any commercial or financial relationships that could be construed as a potential conflict of interest.

## Publisher's Note

All claims expressed in this article are solely those of the authors and do not necessarily represent those of their affiliated organizations, or those of the publisher, the editors and the reviewers. Any product that may be evaluated in this article, or claim that may be made by its manufacturer, is not guaranteed or endorsed by the publisher.

## Supplementary Material

Supplementary Figure 1 The mRNA data characteristics. **(A)** Principal component analysis (PCA). **(B,C)** Distribution of the mRNA abundance in Duroc and Luchuan. Genes were classified based on their rounded log_2_(FPKM) abundance. Each bar represents the gene count as detected by RNA-seq in each category. **(D)** The intersection of genes expressed in Duroc and Luchuan. **(E)** Correlation between Duroc abundance and Luchuan abundance.
